# Differentiated Human Midbrain-Derived Neural Progenitor Cells Express Excitatory Strychnine-Sensitive Glycine Receptors Containing α2β Subunits

**DOI:** 10.1371/journal.pone.0036946

**Published:** 2012-05-11

**Authors:** Florian Wegner, Robert Kraft, Kathy Busse, Wolfgang Härtig, Jörg Ahrens, Andreas Leffler, Reinhard Dengler, Johannes Schwarz

**Affiliations:** 1 Department of Neurology, Hannover Medical School, Hannover, Germany; 2 Carl-Ludwig-Institute of Physiology, University of Leipzig, Leipzig, Germany; 3 Department of Neurology, University of Leipzig, Leipzig, Germany; 4 Department of Neurophysiology, Paul Flechsig Institute of Brain Research, University of Leipzig, Leipzig, Germany; 5 Department of Anaesthesiology and Intensive Care, Hannover Medical School, Hannover, Germany; University of Nebraska Medical Center, United States of America

## Abstract

**Background:**

Human fetal midbrain-derived neural progenitor cells (NPCs) may deliver a tissue source for drug screening and regenerative cell therapy to treat Parkinson’s disease. While glutamate and GABA_A_ receptors play an important role in neurogenesis, the involvement of glycine receptors during human neurogenesis and dopaminergic differentiation as well as their molecular and functional characteristics in NPCs are largely unknown.

**Methodology/Principal Findings:**

Here we investigated NPCs in respect to their glycine receptor function and subunit expression using electrophysiology, calcium imaging, immunocytochemistry, and quantitative real-time PCR. Whole-cell recordings demonstrate the ability of NPCs to express functional strychnine-sensitive glycine receptors after differentiation for 3 weeks *in vitro*. Pharmacological and molecular analyses indicate a predominance of glycine receptor heteromers containing α2β subunits. Intracellular calcium measurements of differentiated NPCs suggest that glycine evokes depolarisations mediated by strychnine-sensitive glycine receptors and not by D-serine-sensitive excitatory glycine receptors. Culturing NPCs with additional glycine, the glycine-receptor antagonist strychnine, or the Na^+^-K^+^-Cl^−^ co-transporter 1 (NKCC1)-inhibitor bumetanide did not significantly influence cell proliferation and differentiation *in vitro.*

**Conclusions/Significance:**

These data indicate that NPCs derived from human fetal midbrain tissue acquire essential glycine receptor properties during neuronal maturation. However, glycine receptors seem to have a limited functional impact on neurogenesis and dopaminergic differentiation of NPCs *in vitro*.

## Introduction

Glycine is an important inhibitory neurotransmitter in the adult central nervous system acting through ionotropic glycine receptors that are most prominently expressed in the brainstem and spinal cord [1]–[3]. These receptors belong to the superfamily of Cys-loop receptors such as GABA_A_, nicotinic acetylcholine, and 5-HT_3_ receptors [4]. As other members of this Cys-loop family, glycine receptors form homomeric or heteromeric pentamers with each of the five subunits arranged around a central ion-conducting pore [5]–[7]. Similar to GABA_A_ receptors in the adult central nervous system, the strychnine-sensitive glycine receptors are involved in regulating inhibitory chloride influx to stabilise the resting membrane potential of neurons. In humans, only four functional glycine receptor subunits have been identified, α1-3 and β [8], which are likely to exist in heteromeric αβ combinations [3]. The α4 subunit gene is a pseudo-gene in humans and there is little evidence for its functional expression in rats [7], [9]. Besides inhibitory glycine receptors, a strychnine-insensitive excitatory glycine receptor contains NMDA receptor subunits from the NR3 family [10].

Defects in glycinergic neurotransmission can result in the neurological motor disorder hyperekplexia which is characterised by a disinhibited startle response [11], [12]. This primary startle syndrome is mostly autosomal dominant and is caused by mutations in the glycine transporter, the α1 subunit, or less frequently by mutations in the β subunit [13]–[16]. However, the genetic basis of many cases of hyperekplexia and paroxysmal movement disorders remains unresolved [17]. Glycine receptors containing the α3 subunit in the spinal cord have been recognised as therapeutic target to treat inflammatory pain syndromes [18].

Glycine receptors play a major role in the excitability of spinal cord and brain stem neurons. During development, the receptor properties undergo molecular changes resulting in modifications of their physiological function. In rats, a developmental switch from α2 homomeric glycine receptors to α1β heteromeric subtypes occurs between birth and the third postnatal week [7]. The knock-out of the α2 subunit, however, has no obvious effect on the behavioural phenotype and neuronal development although it eliminates a tonic glycine-gated chloride current in mouse embryonic cortical neurons [19]. The receptor subtype expression in mouse spinal neurons during *in vitro* development switches similarly from a monomeric α subunit or heteromeric α2β in immature neurons to an α1β isoform in mature neurons. Furthermore, the formation of postsynaptic glycine receptor clusters as well as the receptor affinity to glycine, strychnine, and Zn^2+^ increases during development [20].

In contrast to the adult central nervous system, a high expression of the Na^+^-K^+^-Cl^−^ co-transporter 1 (NKCC1, a Cl^−^ importer) and a low expression of the K^+^-Cl^−^ co-transporter 2 (KCC2, a Cl^−^ exporter) in neural progenitors and immature neurons determine a high intracellular Cl^−^ concentration leading to GABA-induced depolarisations [21]–[24]. Glycine-induced activation of strychnine-sensitive glycine receptors can also lead to hyper- or depolarising responses of the target cells depending on the intracellular Cl^−^ concentration [7]. During neocortical development a depolarising glycine-gated Cl^−^ efflux stimulates the calcium influx [25] necessary for the development of numerous neuronal specialisations including glycinergic synapses [26]. However, the involvement of glycine receptors in human neurogenesis and dopaminergic differentiation as well as their molecular and functional characteristics in human neural progenitor cells (NPCs) are largely unknown.

The proliferation and differentiation of NPCs enables to study human neurogenesis *in vitro* [24], [27]–[32] which shall help to translate neural stem cell therapy for neurodegenerative diseases [33]–[35]. Long-term expanded human mesencephalic NPCs maintain their proliferative capacity and continue to give rise to neurons that express tyrosine hydroxylase (TH) and also release dopamine [36]. The present study analyses human midbrain-derived NPCs in respect to their glycine receptor expression and function. We also investigated whether glycine, the glycine-receptor antagonist strychnine, or the NKCC1-inhibitor bumetanide are able to influence neurogenesis and dopaminergic differentiation *in vitro.*

## Materials and Methods

### Cell Culture

Human neural progenitor cells were derived from CNS tissue of aborted human fetuses (gestational week 10–16) with the informed written consent of all mothers involved in this study. All experiments were approved by the Ethics Committees of the University of Leipzig and the Hannover Medical School, Germany and are in accordance with all state and federal guidelines. The human fetal tissue was washed with sterile Hank’s buffered salt solution and dissected into mesencephalic and non-mesencephalic primary tissue samples. The tissue samples were mechanically separated into small pieces, incubated in 0.1 mg/ml papain solution (Roche, Mannheim, Germany), supplemented with 10 µg/ml DNase (Roche) for 30 min at 37°C, then washed three times with Hank’s buffered salt solution followed by an incubation with 50 µg/ml antipain solution (Roche) for 30 min at 37°C. After three further washing steps the samples were homogenised by gentle trituration using fire-polished pasteur pipettes. The quality of the tissue was assessed as described previously [27], [28].

Propagation of human mesencephalic neural progenitor cells (NPCs) was performed in a monolayer by plating onto poly-L-ornithine (Sigma, Taufkirchen, Germany) and fibronectin (Millipore, Billerica, MA, USA) coated culture dishes at a density of 30000 cells/cm^2^. For expansion of NPCs, we used a xeno-free medium (Dulbecco’s modified Eagle’s medium/F12) supplemented with epidermal growth factor and basic fibroblast growth factor (20 ng/ml each; both from PromoCell, Heidelberg, Germany), 2% B27 (Invitrogen, Karlsruhe, Germany), and 1% penicillin/streptomycin (PAA, Pasching, Austria). Cells could be expanded for prolonged periods (>10 passages) in reduced atmospheric oxygen (3%) as described previously [36]–[38]. For differentiation NPCs were plated on poly-L-lysine coated 35 mm dishes (Sigma) at a density of 1×10^5^/cm^2^. Neuronal differentiation was induced by replacement of the expansion medium by a mitogen-free standard medium consisting of Neurobasal (Invitrogen) supplemented with 2% B27 (Invitrogen), 1% Glutamax, interleukin-1β (100 pg/ml; Sigma), 5 µM forskolin (Sigma), and 0.1% gentamycin (Invitrogen). The differentiation medium was replaced twice a week during the whole incubation protocol. Note that the expansion medium contained 250 µM glycine (Sigma) and the standard differentiation medium 400 µM glycine.

### Electrophysiology

Whole-cell patch-clamp recordings of ligand- and voltage-gated ion channels were performed on human midbrain-derived NPCs that had been differentiated for 3 weeks *in vitro.* Experiments were carried out in the voltage- or current-clamp mode (holding potential −70 mV) at room temperature using an EPC-9 amplifier and PulseFit software (HEKA, Lambrecht, Germany). The external bath solution contained (in mM): 142 NaCl, 1 CaCl_2_, 8 KCl, 6 MgCl_2_, 10 glucose, and 10 HEPES (pH 7.4; 320 mOsm). Micropipettes were formed from thin-walled borosilicate glass (BioMedical Instruments, Zöllnitz, Germany) with a Flaming Brown electrode puller P-97 (Sutter Instrument Co., Novato, CA, USA) and a Micro Forge (Narishige, Tokio, Japan). Electrodes had resistances of 3–5 MΩ when filled with the internal solution containing (in mM): 153 KCl, 1 MgCl_2_, 10 HEPES, 5 EGTA, and 2 MgATP (pH 7.3; 305 mOsm). The combination of internal and external solutions produced a chloride equilibrium potential near 0 mV for glycine receptor recordings.

All solvents and chemicals for pharmacological experiments were purchased from Sigma or Tocris (Germany). The stock solutions were prepared in DMSO or external recording solution as appropriate (1–300 mM). A fresh stock solution of tropisetron (1 mM) was prepared at the day of experiments. The drugs were dissolved in external solution containing DMSO at a maximal final concentration of 0.1%. All drugs were applied rapidly via gravity using a modified SF-77B perfusion fast-step system (Warner Instruments Inc., Hamden, CT, USA) as described previously [39]. For the glycine dose-response curve seven increasing concentrations (10 µM–10 mM) were applied for 2 sec on NPCs. For pharmacological characterisation of glycine receptors, positive and negative modulators were co-applied for 2 sec with an EC_70_ of glycine (300 µM). The intervals between applications were 30 sec, after co-applying strychnine 1 min intervals were allowed for wash out.

**Table 1:** Functional properties of human mesencephalic NPCs after differentiation for 3 weeks *in vitro*.

Functional properties of differentiated NPCs	Glycine-responsive NPCs (n = 53)	Glycine-nonresponsive NPCs (n = 26)
Peak Na^+^-current	−73±12 pA	−120±23 pA *
Peak Na^+^-current/pF	8.4±1.7 pA/pF	10.6±2.2 pA/pF
Peak K^+^-current	948±107 pA	1246±131 pA
Peak K^+^-current/pF	106±11 pA/pF	108±11 pA/pF
Membrane capacitance	9.1±0.6 pF	12.0±0.9 pF **
Membrane potential	−30.2±2.5 mV	−33.9±3.0 mV
Input resistance	470±53 MΩ	648±119 MΩ

Voltage-gated currents and passive membrane properties of NPCs displaying currents during application of glycine (glycine-responsive) are shown in comparison to cells without detectable glycine-induced current (<5 pA, nonresponsive). All data are given as means ± SEM (*p<0.05, **p<0.01, t-test).

**Table 2:** Quantitative real-time PCR analysis of glycine receptor subunit expression in human mesencephalic NPCs after 1 and 3 weeks of differentiation.

Glycine receptor subunit	Sequence (forward; reverse)	Product (bp)	ΔCt values (3 weeks differentiation)	ΔCt values (1 week differentiation)
α1	TAAGGAGGCTGAAGCTGCTC; ATGTTGCAGCTCACGTTCAC	144	16.0±1.2	13.1±0.8
α2	ACGATGACCACCCAGAGTTC; CCAGTAAGGCAGCAAACACA	115	6.3±0.2 *	10.4±1.2
α3	TAAGCCATTCGCAAGATGTG; TTTGCACCCAATTACACTGC	130	10.0±1.3	10.1±0.5
α4	CTCACCATGACCACCCAGA; GCGAACACAAAGAGCAGACA	107	12.5±0.5 *	17.7±1.2
β	CACATGCGTGGAAGTCATCT; GGAAAGCCAGGAGAGAACAA	93	5.4±0.03 *	7.8±0.6

Primer sequences, amplification product in base pairs, and mean ΔCt values ± SEM (n = 3) are given for each investigated receptor subunit (*p<0.05, t-test). Note, a low ΔCt value represents a high expression level.

Whole-cell currents were low-pass filtered at 1–5 kHz, digitized at 10 kHz, and analysed with PulseFit (HEKA) and GraphPad Prism (GraphPad Software, San Diego, CA, USA). Peak currents of each investigated cell were normalised to the maximal glycine-evoked peak current (for glycine dose-response curves) or to the glycine EC_70_ control that was applied prior to the co-application of each tested modulator. To obtain nonlinear regression concentration-response plots mean peak currents ± SEM were fitted to a sigmoidal function using a four parameter logistic equation (sigmoidal concentration-response) with a variable slope. The equation used to fit the concentration-response relationship was I = I_max_/1+10^(LogEC50−Logdrug)xHill slope^ where *I* was the peak current at a given concentration. Numerical data of all experiments were expressed as means ± SEM. Statistical differences were calculated by using Student’s t test (two tailed, unpaired) and considered significant at p<0.05 (Table 1).

### Quantitative Real-time PCR

After 1 and 3 weeks of differentiation *in vitro*, human mesencephalic NPCs from 3 cell lines were harvested and total RNA isolated using the Trizol reagent (Invitrogen). Reverse transcription of 800 ng total RNA per reaction was carried out using oligo-dT primer and Superscript II reverse transcriptase (Invitrogen).

Quantitative real-time PCR was done using cDNA from 30 ng total RNA, 0.6 µM forward and reverse primers, Platinum-SYBR Green® qPCR Supermix (Invitrogen), and 100 nM 6-carboxy-X-rhodamine (ROX) using the following protocol in an MX 3000P instrument (Stratagene, La Jolla, CA, USA): 2 min 50°C, 2 min 95°C and 50 cycles of 15 sec 95°C, 30 sec 60°C. To confirm a single amplicon a product melting curve was recorded. The correct amplicon size was asserted by agarose gel electrophoresis using low molecular weight DNA ladder (New England Biolabs, Ipswich, MA, USA). Oligonucleotide primers for human glycine receptor subunits α1–4 and β (Table 1) were designed to flank intron sequences, if feasible, using Primer 3 software.

Cycle threshold (Ct) values were placed within the exponential phase of the PCR as described previously [40]. Ct values of 3 independent experiments, each performed in duplicate, were normalised to β2-microglobulin (Ct – Ct β2-microglobulin = ΔCt). ΔCt values were used to calculate the relative subunit expression levels and are given as means ± SEM (Table 2). Note, a low ΔCt value represents a high expression level. The expression of glycine receptor subunits was statistically evaluated by subjecting ΔCt values to a one-way ANOVA and Newman-Keuls post test for multiple comparisons taking statistical significance as p<0.05.

### Calcium Imaging

Measurements of the intracellular Ca^2+^ concentration [Ca^2+^]_i_ in human mesencephalic NPCs were performed as described previously [24] using a monochromator-based imaging system (TILL Photonics, Gräfelfing, Germany). Cells were loaded with 5 µM fura-2-AM (Invitrogen) supplemented with 0.01% Pluronic F127 for 30 min at 20–22°C in a standard bath solution containing (mM): 140 NaCl, 5 KCl, 2 CaCl_2_, 10 glucose and 10 HEPES, adjusted to pH 7.4 with NaOH. For measurements of [Ca^2+^]_i_, cells were placed in a recording chamber and continuously perfused with standard bath solution at a rate of 5 ml/min. Fluorescence was excited at 340 and 380 nm and emitted fluorescence intensities from single cells were acquired at intervals of 2 s.

**Figure 1:**
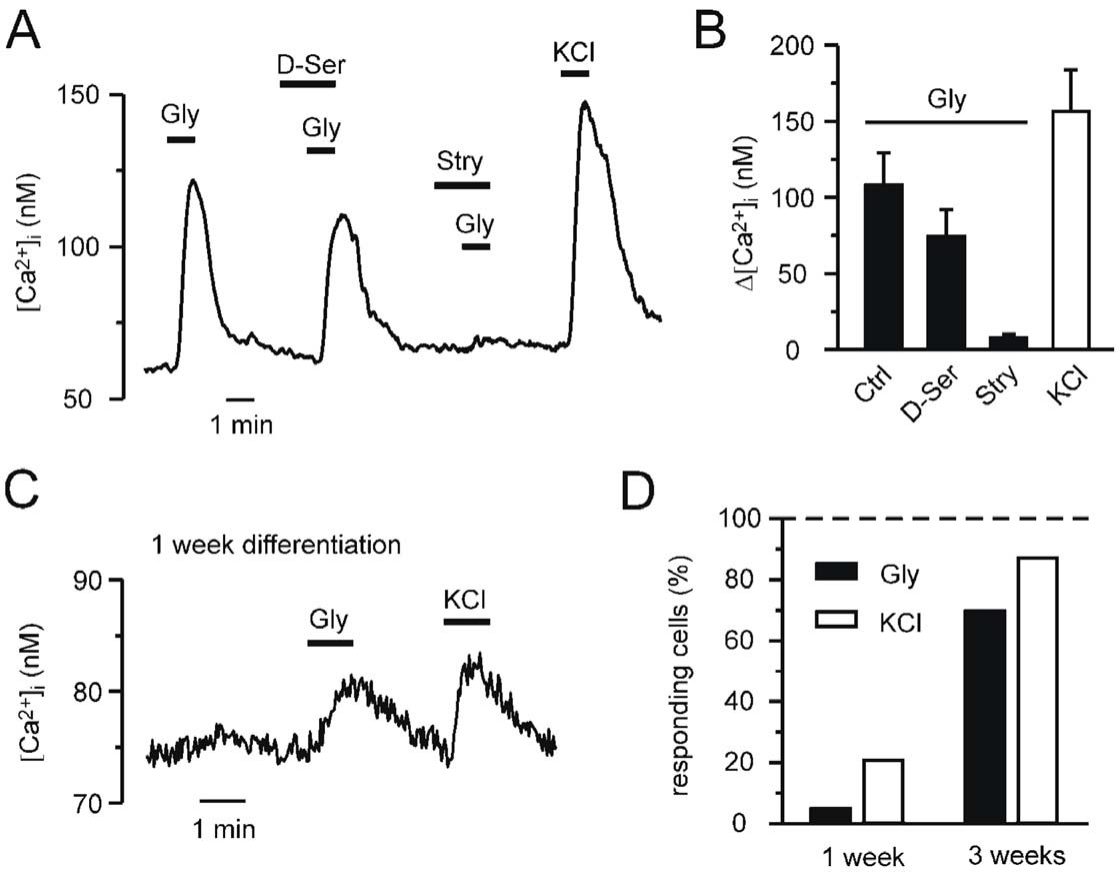
Glycine-induced Ca^2+^ signalling in fura-2 loaded NPCs differentiated for 1 or 3 weeks. A, Changes in [Ca^2+^]_i_ were evoked by application of 100 µM glycine (Gly), 100 µM D-serine (D-Ser), 20 µM strychnine (Stry), and 50 mM KCl in cells differentiated for 3 weeks. The trace is the mean response of eight cells from a representative experiment. B, Summary of the [Ca^2+^]_i_ response amplitudes (n = 22–27 cells; means ± SEM) and fractions of cells (n = 60–75 from 86 cells) responding to different stimuli was obtained from 4 experiments as shown in A. C, NPCs differentiated for 1 week showed smaller increases in [Ca^2+^]_i_ upon application of 100 µM glycine and 50 mM KCl. The trace is the mean response of 8 cells from a representative experiment. D, Fractions of cells differentiated for 1 week (n = 7–29 of 139 cells) or 3 weeks (n = 60–75 from 86 cells) responding to different stimuli were obtained from 5 and 4 experiments as shown in A and C, respectively.

### Drug Treatment of NPCs

To investigate the influence of additional glycine, the glycine-receptor antagonist strychnine, and the NKCC1-inhibitor bumetanide on NPCs *in vitro*, separate experiments were performed in which some cells were treated with two distinct concentrations of glycine (1 and 10 mM), strychnine, or bumetanide (1 and 10 µM) during proliferation (2 weeks) and differentiation (1 and 3 weeks). The stock solution was prepared by dissolving glycine in distilled water at a concentration of 100 mM, strychnine in ethanol and bumetanide in DMSO at a concentration of 10 mM. The drugs were renewed with every media change. Besides using the standard differentiation medium, NPCs were also differentiated for 1 week by a novel mitogen-free medium to promote dopaminergic neurogenesis consisting of Neurobasal (Invitrogen) supplemented with 2% B27 (Invitrogen), 1% Glutamax, 100 µM dbcAMP (Sigma), 10 µM forskolin (Sigma), 100 µM fusaric acid (Sigma), and 0.1% gentamycin (Invitrogen).

The determination of cell number and protein content as well as the Western blotting of treated and untreated NPCs were performed as described previously [27], [28]. Primary antibodies used for Western blotting were as follows: mouse monoclonal anti-PCNA (proliferating cell nuclear antigen; Santa Cruz, Heidelberg, Germany), mouse monoclonal anti-Bcl-2 (B cell lymphoma 2; Santa Cruz), mouse monoclonal anti-actin (MP Biomedicals, Eschwege, Germany), rabbit polyclonal anti-ß-tubulin III (Covance, Freiburg, Germany), rabbit polyclonal anti-TH (Santa Cruz, Heidelberg, Germany), and rat anti-GFAP (glial fibrillary acidic protein; Zymed, San Francisco, CA, USA).

**Figure 2:**
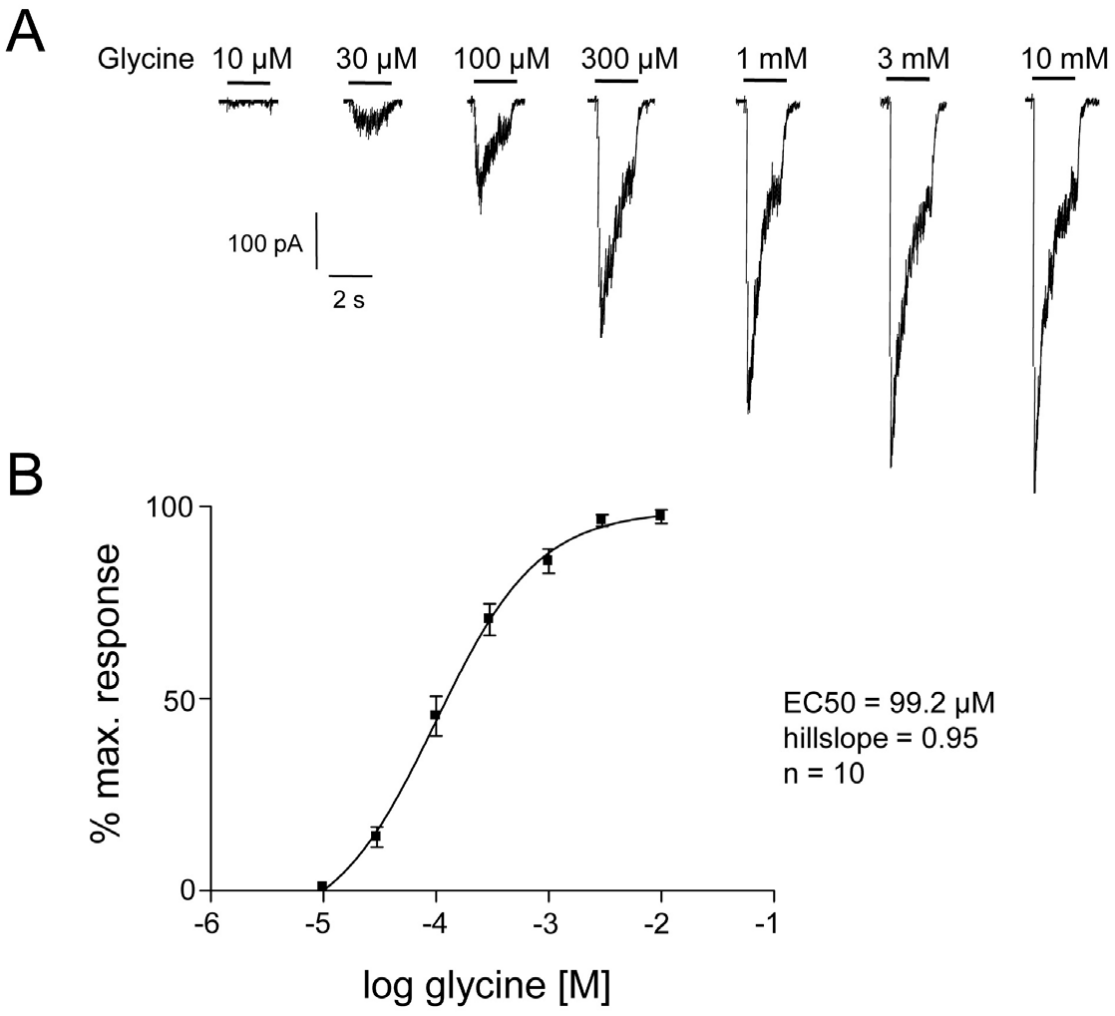
Functional analysis of glycine receptors in human midbrain-derived NPCs differentiated for 3 weeks *in vitro*. A, Whole-cell recordings of currents elicited by applications of increasing glycine concentrations (10 µM – 10 mM). B, Glycine dose-response curve (EC_50_ = 99.2 µM, hillslope = 0.95, n = 10) indicates a glycine EC_70_ of 300 µM that was used in further electrophysiological experiments.

**Figure 3:**
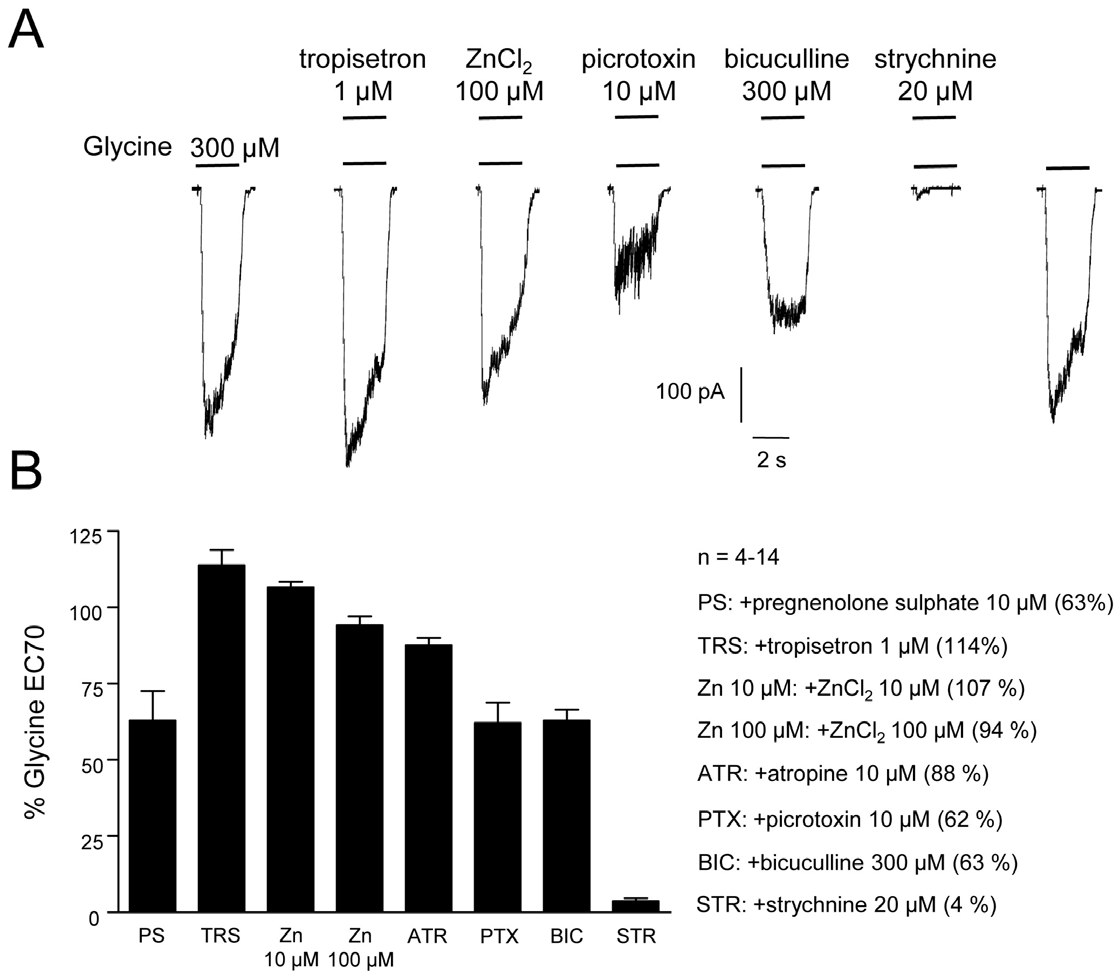
Pharmacological characterisation of glycine receptors in NPCs differentiated for 3 weeks. A, Whole-cell currents evoked by a glycine EC_70_ were all markedly blocked by strychnine and partly inhibited by picrotoxin, pregnenolone sulphate and a high bicuculline concentration. In contrast to the positive modulation by 10 µM ZnCl_2_, a reduction of glycine currents was induced by 100 µM ZnCl_2_. Also, co-application of a glycine EC_70_ and tropisetron showed a positive modulatory effect. This pharmacological profile suggests receptor isoforms containing α2β subunits (B, n = 9–10; all data are given as means ± S.E.M.).

**Figure 4:**
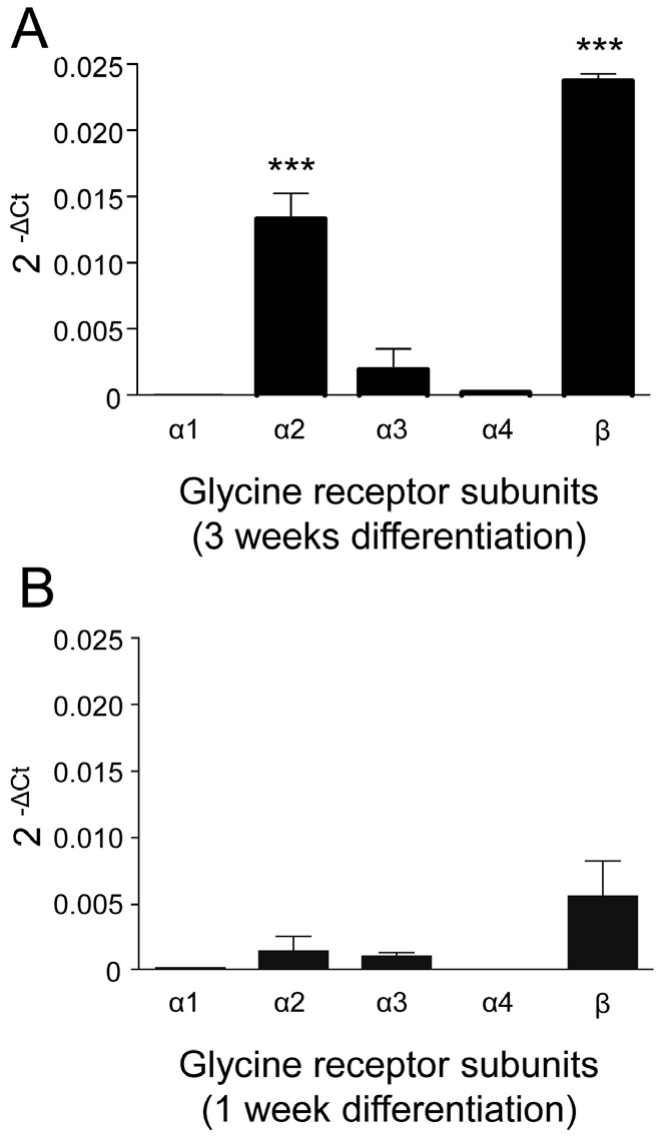
Real time PCR analysis of glycine receptor subunits expressed by NPCs after 1 and 3 weeks of differentiation. Quantitative real-time PCR (SYBR green assay) was performed for each transcript and control (β2-microglobulin). Cycle threshold (Ct) values were normalized to the Ct values of the control and are given as log2^−ΔCt^ (ΔCt = Ct – Ct β2-microglobulin). Data are presented as means ± S.E.M. of 3 independent experiments (3 NPC lines) each performed in duplicate. A, The bar graph shows predominant expression of α2 and β subunits in NPCs differentiated for 3 weeks that is significantly different from all other glycine receptor subunits; significant differences between log2^−ΔCt^ values are marked (***p<0.001, ANOVA and Newman-Keuls post test). B, After 1 week of cell maturation the various glycine receptor isoforms are not yet expressed to a significantly different extent. The expression of α2, α4 and β subunits was markedly lower in NPCs differentiated for 1 week than for 3 weeks.

**Figure 5:**
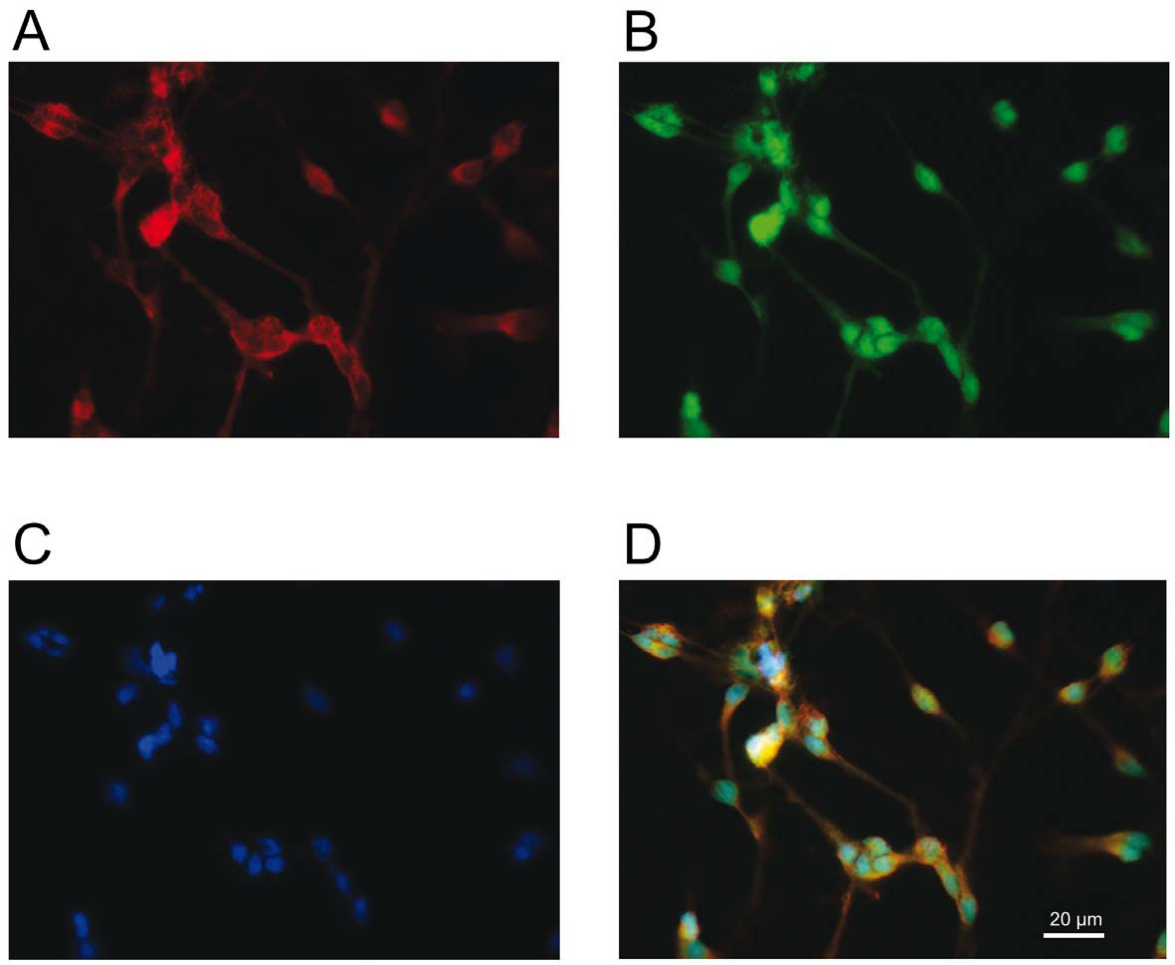
Immunocytochemistry of human mesencephalic NPCs after 3 weeks of standard differentiation in vitro. Photomicrographs of NPCs immunoreactive for MAP2 (A) and glycine receptor subunits (B); nuclei were counter-stained with DAPI (C). Merged picture illustrates that neuronal cells express glycine receptor subunits (D). Note, this glycine receptor antibody is specific for all subunits.

### Immunocytochemistry

For immunolabelling, differentiated cells were fixed with 4% paraformaldehyde for 10 min. The unspecific binding was blocked in PBS supplemented with 10% fetal calf serum and membranes were permeabilized with 0.3% Triton X-100 for 1 h. Cultures were incubated with primary antibodies for 2 h at room temperature. The following primary antibodies were used for immunofluorescence analysis: rabbit polyclonal anti-TH (Santa Cruz) diluted 1∶500, mouse monoclonal anti-β-tubulin III (1∶500, Sigma), mouse monoclonal anti-MAP2 (1∶300, Pharmingen, Heidelberg, Germany), rat monoclonal anti-GFAP (1∶500, Zymed). The cells were incubated for 1 h at room temperature with fluorescent secondary antibodies conjugated either to Alexa Fluor 488 or Alexa Fluor 594 (1∶500, Invitrogen). Nuclei were stained with 4′,6-diamidino-2-phenylindole (DAPI, 0.5 mg/ml, Calbiochem, San Diego, CA, USA) for 30 min at room temperature.

For immunocytochemical staining of glycine receptor subunits in NPCs after 3 weeks of standard differentiation, we used a modified protocol. Unspecific binding was blocked with 5% donkey serum (Dianova, Hamburg, Germany) and membranes were permeabilized with 0.3% Triton X-100 for 1 h. The primary antibodies (mouse monoclonal anti-glycine receptor, mAb4a, 1∶250, Synaptic Systems, Goettingen, Germany; rabbit polyclonal anti-MAP2, AB5622, 1∶500, Chemicon, Nuernberg, Germany) were incubated in PBS/5% donkey serum/0.3% Triton X-100 for 20 h at 4°C. Note, this glycine receptor antibody is specific for all subunits. Cultures were incubated in 2% bovine serum albumin with secondary antibodies (Alexa Fluor 488, 1∶500, Invitrogen; red fluorescent indocarbocyanine Cy3, 1∶ 200, Dianova) for 2 h at room temperature. Nuclei were counter-stained as described above.

Staining patterns were visualised by fluorescence microscopy (Axiovert 200, Zeiss, Jena, Germany). Digital images were acquired with an AxioCam MRc camera using the image-analysis software AxioVision 4 (Zeiss). The portion of cells immunoreactive for GFAP, β-tubulin III, MAP2, and TH was determined by counting the number of immunopositive cells in relation to the number of DAPI stained nuclei. Approximately 1000 cells were counted within four randomly selected fields per well.

## Results

We studied the functional and molecular glycine receptor properties of human midbrain-derived NPCs following differentiation using standard conditions for 1 or 3 weeks *in vitro*. The effect of glycine on Ca^2+^ signalling in differentiated mesencephalic NPCs was determined using fura-2-based Ca^2+^ imaging. Besides its agonistic action on glycine receptors, glycine is also a co-agonist along with glutamate for most NMDA receptors and can activate NR3 subunit containing NMDA receptors in the absence of glutamate [10]. To test for a depolarisiation-induced entry of Ca^2+^ due to activation of glycine receptors as well as for a possible involvement of glycine-activated NMDA receptors, we subsequently applied glycine alone or in combination with the antagonists strychnine or D-serine. The latter, a co-agonist of conventional NMDA receptors, was shown to inhibit currents of NMDA receptors containing NR3 [10].

NPCs differentiated for 3 weeks showed glycine-induced Ca^2+^ transients that were completely suppressed by the glycine receptor antagonist strychnine but were only slightly affected by D-serine (Fig. 1A). Increases in [Ca^2+^]_i_ were also evoked by application of 50 mM KCl, indicating depolarisation-dependent Ca^2+^ entry. The percentage of cells responding to glycine and KCl determined from all experiments was 70% (n = 60 from 85) and 87% (n = 75 from 85), respectively (Fig. 1B). The increases in [Ca^2+^]_i_ elicited by subsequent application of 100 µM glycine, of 100 µM glycine in the presence of either D-serine or strychnine, and of 50 mM KCl were 108±21 nM (n = 22), 74±17 nM (n = 22), 8±2 nM (n = 22), and 157±27 nM (n = 29), respectively (Fig. 1B). These data suggest that glycine evokes Ca^2+^ signals by activation of glycine receptors and receptor-dependent membrane depolarisation. Human midbrain-derived NPCs differentiated for 1 week were less responsive to application of glycin and KCl (Fig. 1C). The percentages of cells responding to glycine determined from these experiments were 5% (n = 7 of 139) and 70% (n = 60 of 85) for shorter (1 week) and longer (3 weeks) differentiation, respectively (Fig. 1D). The corresponding amounts of cells showing KCl-induced Ca^2+^ signals were 21% (n = 29 of 139) and 87% (n = 75 of 85) for shorter and longer differentiation, respectively (Fig. 1D). We also tested the ability of glycine to induce [Ca^2+^]_i_ changes in undifferentiated NPCs. However, all tested cells displayed no measurable responses to application of 100 µM glycine (n = 60 from 4 experiments, data not shown).

Patch-clamp electrophysiology was performed to measure glycine-evoked currents as well as voltage-gated sodium and potassium currents. Whole-cell recordings revealed a current response during rapid applications of glycine in 67% of differentiated NPCs (n = 53 from 79), which is similar to the percentage of glycine-responsive cells in calcium imaging experiments. The average peak sodium currents of glycine-responsive NPCs (–73 pA) were significantly smaller in comparison to cells without detectable glycine-induced current (–119 pA; Table 1). However, NPCs expressing functional glycine receptors showed a smaller cell membrane capacitance than cells without current evoked by glycine (9.1 pF and 12.0 pF, respectively; Table 1). Therefore, the relative sodium and potassium peak current densities in pA/pF were not significantly different between these groups of NPCs. Furthermore, there were no significant differences for peak potassium currents, resting membrane potentials, and input resistances between both cell types (Table 1).

Rapid applications of increasing glycine concentrations (10 µM–10 mM) on differentiated NPCs elicited desensitising currents in a dose-dependent manner (Fig. 2A). The glycine concentration-response plot (Fig. 2B) indicated an EC_50_ of 99.2 µM (95% confidence interval of Log EC_50_ value –4.181 to –3.826, hillslope 0.95, n = 10). Mean peak currents evoked by glycine were 241 pA±41 pA (n = 53). For further pharmacological characterisation of glycine receptors, we used co-applications of 300 µM glycine corresponding to the EC_70_ according to the concentration-response plot.

All glycine-induced currents of NPCs were markedly blocked by strychnine and partly inhibited by atropine, the neurosteroid pregnenolone sulphate as well as by the GABA_A_ receptor antagonists picrotoxin and bicuculline (Fig. 3A). In contrast to the slight inhibition of glycine currents by 100 µM Zn^2+^, a small positive modulatory effect was induced by a lower Zn^2+^−concentration (10 µM; Fig. 3B). Furthermore, a current potentiation of the glycine EC_70_ was induced by co-application of the 5-HT3-receptor antagonist tropisetron. The pharmacological profile of glycine receptors in NPCs with moderate picrotoxin-sensitivity, a differential sensitivity to Zn^2+^, and a positive modulation by tropisetron, suggests the expression of heteromeric isoforms, most likely containing α2β subunits [41], [42].

For quantitative expression analysis of glycine receptor subunits, human midbrain-derived NPC lines (n = 3) were investigated by real-time PCR after differentiation for 1 and 3 weeks *in vitro*. Statistical comparisons of ΔCt values (n = 3 independent experiments, each performed in duplicate) are summarised in Fig. 4. While the expression of various glycine receptor subunits was not yet markedly different after 1 week of cell maturation (Fig. 4B), we found a predominant expression of α2 and β subunits with significant difference to the other glycine receptor isoforms after differentiation for 3 weeks (Fig. 4A; p<0.001, ANOVA and Newman-Keuls post test). The expression of α2, α4 and β subunits was significantly higher in NPCs differentiated for 3 weeks than for 1 week (Table 2; p<0.05, t-test). Interestingly, β subunit expression was most pronounced and significantly different from all α subunits after 3 weeks of maturation (Fig. 4A). The expression of α1, which is the most abundant glycine receptor α subunit in the adult nervous system, was barely detectable, whereas the α4 subunit sparsely occurred and α3 was expressed to a moderate extent. In line with the pharmacological results, the quantitative PCR data indicate a predominant expression of glycine receptors containing α2 and β subunits.

Drug treatment of human mesencephalic NPCs with additional glycine (1 and 10 mM), the glycine-receptor antagonist strychnine, or the NKCC1-inhibitor bumetanide (1 and 10 µM) during expansion (2 weeks) did not induce significant differences compared to untreated controls (n = 3–6) in the number of living cells, protein content, or the cell proliferation as determined by Western blot analysis of the proliferation marker PCNA and the survival marker Bcl2 as well as by immunocytochemistry of the proliferation marker Ki67 and the neural stem cell marker nestin (data not shown). After differentiation with the standard protocol (1 and 3 weeks) or with a novel medium to promote the dopaminergic neurogenesis (1 week), Western blot and immunocytochemical results of drug treated NPCs (n = 3–6 cell lines) using the markers GFAP, β-tubulin III, MAP2, and TH were not significantly different from controls (data not shown).

The amount of untreated NPCs (n = 3 cell lines) immunopositive for TH (0.9%), β-tubulin III (34.4%), and GFAP (31.3%) after 1 week increased moderately during a total differentiation of 3 weeks with the standard protocol to 1.6%, 39.6%, and 32.6%, respectively. The number of MAP2-immunoreactive NPCs was significantly higher after 3 weeks (30.5%) than 1 week (13.7%) of maturation (n = 3, p<0.05, t-test). Using a novel medium to enhance dopaminergic neurogenesis of NPCs (n = 6 cell lines) resulted in a marked increase of TH-immunoreactive cells to 2.7% after differentiation for 1 week. Immunocytochemical stainings after 3 weeks of standard NPC-differentiation show that neuronal MAP2-expressing cells are immunopositive for glycine receptor subunits (Fig. 5).

## Discussion

In this study, calcium imaging and electrophysiological recordings demonstrated the expression of excitatory strychnine-sensitive glycine receptors in differentiated human midbrain-derived NPCs. The similar voltage-gated peak currents/pF in cells with functional glycine receptors (67%) compared to glycine-nonresponsive NPCs (33%, Table 1) suggest that the maturation of functional glycine receptor properties and voltage-gated channels is not a simultaneous process contrasting GABA_A_ receptor expression in these cells [24]. Previously, we could show that GABA induces [Ca^2+^]_i_ increases in NPCs due to membrane depolarisation mediated by GABA_A_ receptors [24]. Activation of glycine receptors corresponded with depolarising effects leading to a similar rise of [Ca^2+^]_i_ in NPCs which could be completely inhibited by co-application of strychnine. Calcium responses in NPCs revealed only a weak sensitivity of excitatory glycine receptors to D-serine that blocks glycine-evoked currents at NMDA receptors containing NR3 subunits [10]. Although human mesencephalic NPCs express low amounts of NR3 [32], our calcium imaging data suggest that glycine induces depolarisations by activation of strychnine-sensitive glycine receptors.

Previous approaches to analyse human fetal neural progenitors revealed intracellular Ca^2+^ responses to glycine with a lower magnitude and frequency (6%) after 2 to 4 weeks of differentiation [43], [44] suggesting an immature state of glycine receptor expression and function. Furthermore, a subpopulation of human corneal stem cells responded to glycine in electrophysiological experiments [45]. Strychnine-sensitive glycine receptors with a depolarising function were detected indirectly by glycine-induced suppression of inwardly-rectifying potassium channels in 80% of neural stem-like cells derived from human umbilical cord blood [46]. However, the functional relevance of glycine receptor-mediated depolarisations for neurogenesis remains unclear. High levels of glycinergic transmission may modulate neuronal excitability causing membrane depolarisation and changes in intracellular calcium that possibly regulate gene activity and affect neurite outgrowth in immature mouse spinal neurons [20].

In differentiated human mesencephalic NPCs, the pronounced expression of the importing Cl^−^ co-transporter NKCC1 contrasts KCC2 expression [24] which can result in a high intracellular Cl^−^ concentration and depolarising glycine-effects. Neuronal maturation is associated with a downregulation of NKCC1 and a stronger appearance of KCC2 [21]–[23] that changes the direction of the ligand-gated Cl^−^ current at strychnine-sensitive glycine receptors from a depolarising to a hyperpolarising one. We intended to test the role of glycine receptors and NKCC1 for proliferation and differentiation of NPCs *in vitro* by culturing the cells with the NKCC1-inhibitor bumetanide (1 and 10 µM, [23]), the glycine-receptor antagonist strychnine (1 and 10 µM), or additional glycine (1 and 10 mM) while the media contained moderate glycine concentrations (250–400 µM). However, such drug treatment of human mesencephalic NPCs did not reveal significant changes compared to untreated controls regarding markers for neurogenesis and dopaminergic differentiation suggesting that glycine receptors seem to have a limited functional impact on proliferation and maturation of NPCs *in vitro*. The marked increase of MAP2-immunopositive cells between 1 and 3 weeks of differentiation reflects neuronal maturation that is also apparent in glycine receptor function (Fig. 1) and subunit expression (Fig. 4) as well as in voltage-gated channel, GABA_A_- and glutamate receptor function of NPCs [24].

Mature glycine receptors in the adult CNS display molecular structures and physiological properties different from those in the immature CNS. Immature glycine receptors are usually equipped with α2 or α2β subunits while mature receptors are characterized by their content of α1β and an increased sensitivity to several modulators [7], [20]. Most studies using recombinant glycine receptors determined higher EC_50_ values for isoforms containing β subunits than for the corresponding subtypes devoid of β [41], [47]–[49]. The glycine EC_50_ of receptors with α2 or α2β subunits mainly ranged from 62 to 96 µM and from 66 µM to 157 µM, respectively [41], [49]. For the native glycine receptors in NPCs, we calculated an EC_50_ of 99.2 µM. The glycine-induced currents (EC_70_∶300 µM) were all blocked by strychnine and showed a rather low sensitivity to many modulators suggesting immature receptor subtypes. In addition, the results of quantitative PCR indicated a lack of α1 expression and α2 and β as predominant subunits leading to the assumption that NPCs express glycine receptors with α2 and/or α2β subunits.

In pharmacological experiments we tried to distinguish these subtypes, although there are few compounds with sufficient discriminatory capacity to identify the presence of either homomeric α2 or heteromeric α2β glycine receptors [7]. While the glycine responses of recombinant α2 receptors are inhibited by the 5-HT3-antagonist tropisetron [41], [47], the responses of α2β subtypes can be potentiated by low tropisetron concentrations [41]. In human mesencephalic NPCs, co-application of tropisetron (1 µM) enhanced glycine-induced currents indicating the presence of β subunits which are likely to contribute to this modulatory glycine receptor site. A differential sensitivity to Zn^2+^ that can potentiate glycine-evoked currents at lower micromolar concentrations and inhibit recombinant heteromeric and neuronal glycine receptors at higher concentrations [42], [50] could also be demonstrated for the receptor isoforms of NPCs suggesting α2β subunit expression. Picrotoxin and the neurosteroid pregnenolone sulphate are more potent inhibitors at homomeric than at heteromeric glycine receptors [48], [51]–[53]. Similar to recombinant α2β subtypes [41], [48], 10 µM picrotoxin and pregnenolone sulphate caused only a moderate reduction of glycine-evoked currents at native NPC receptors. Corresponding to the quantitative PCR data for human mesencephalic NPCs, the elucidated pharmacological profile indicates the expression of strychnine-sensitive glycine receptors with α2β subunits.

In conclusion, the analyses of NPCs derived from human fetal midbrain tissue demonstrate the expression of strychnine-sensitive glycine receptors with an excitatory function following differentiation but not during proliferation. Molecular and pharmacological evidence suggest a predominant role for heteromeric α2β subtypes which may indicate that cells are not fully maturated. This study suggests that human mesencephalic NPCs acquire essential glycine receptor properties during neuronal differentiation *in vitro*.
